# Comparative diagnostic performance of ultrasound shear wave elastography and magnetic resonance elastography for classifying fibrosis stage in adults with biopsy-proven nonalcoholic fatty liver disease

**DOI:** 10.1007/s00330-021-08369-9

**Published:** 2021-12-02

**Authors:** Yingzhen N. Zhang, Kathryn J. Fowler, Andrew S. Boehringer, Vivian Montes, Alexandra N. Schlein, Yesenia Covarrubias, Tanya Wolfson, Cheng W. Hong, Mark A. Valasek, Michael P. Andre, Rohit Loomba, Claude B. Sirlin

**Affiliations:** 1 Liver Imaging Group, Altman Clinical and Translational Research Institute, Department of Radiology, University of California, San Diego, 9452 Medical Center Drive, La Jolla, CA 92037 USA; 2 Department of Pathology, University of California, San Diego, 9452 Medical Center Drive, La Jolla, CA 92037 USA; 3 Department of Radiology, University of California, San Diego, 9452 Medical Center Drive, La Jolla, CA 92037 USA; 4 NAFLD Research Center, Department of Medicine, University of California, San Diego, 9452 Medical Center Drive, La Jolla, CA 92037 USA; 5 Division of Gastroenterology, Department of Medicine, University of California, San Diego, 9452 Medical Center Drive, La Jolla, CA 92037 USA

**Keywords:** Nonalcoholic fatty liver disease, Elasticity imaging techniques, Sonoelastography, Magnetic resonance elastography

## Abstract

**Objectives:**

To compare the diagnostic accuracy of US shear wave elastography (SWE) and magnetic resonance elastography (MRE) for classifying fibrosis stage in patients with nonalcoholic fatty liver disease (NAFLD).

**Methods:**

Patients from a prospective single-center cohort with clinical liver biopsy for known or suspected NAFLD underwent contemporaneous SWE and MRE. AUCs for classifying biopsy-determined liver fibrosis stages ≥ 1, ≥ 2, ≥ 3, and = 4, and their respective performance parameters at cutoffs providing ≥ 90% sensitivity or specificity were compared between SWE and MRE.

**Results:**

In total, 100 patients (mean age, 51.8 ± 12.9 years; 46% males; mean BMI 31.6 ± 4.7 kg/m^2^) with fibrosis stage distribution (stage 0/1/2/3/4) of 43, 36, 5, 10, and 6%, respectively, were included. AUCs (and 95% CIs) for SWE and MRE were 0.65 (0.54–0.76) and 0.81 (0.72–0.89), 0.81 (0.71–0.91) and 0.94 (0.89–1.00), 0.85 (0.74–0.96) and 0.95 (0.89–1.00), and 0.91 (0.79–1.00) and 0.92 (0.83–1.00), for detecting fibrosis stage ≥ 1, ≥ 2, ≥ 3, and = 4, respectively. The differences were significant for detecting fibrosis stage ≥ 1 and ≥ 2 (*p* < 0.01) but not otherwise. At ≥ 90% sensitivity cutoff, MRE yielded higher specificity than SWE at diagnosing fibrosis stage ≥ 1, ≥ 2, and ≥ 3. At ≥ 90% specificity cutoff, MRE yielded higher sensitivity than SWE at diagnosing fibrosis stage ≥ 1 and ≥ 2.

**Conclusions:**

In adults with NAFLD, MRE was more accurate than SWE in diagnosing stage ≥ 1 and ≥ 2 fibrosis, but not stage ≥ 3 or 4 fibrosis.

**Key Points:**

• *For detecting any fibrosis or mild fibrosis, MR elastography was significantly more accurate than shear wave elastography*.

• *For detecting advanced fibrosis and cirrhosis, MRE and SWE did not differ significantly in accuracy*.

• *For excluding advanced fibrosis and potentially ruling out the need for biopsy*, *SWE and MRE did not differ significantly in negative predictive value*.

• *Neither SWE nor MRE had sufficiently high positive predictive value to rule in advanced fibrosis*.

**Supplementary Information:**

The online version contains supplementary material available at 10.1007/s00330-021-08369-9.

## Introduction

With an estimated global prevalence of 25%, nonalcoholic fatty liver disease (NAFLD) is the most common chronic liver disease worldwide [1]. NAFLD comprises both nonalcoholic fatty liver and nonalcoholic steatohepatitis (NASH), the latter of which is characterized by hepatocellular injury, inflammation, and higher potential for developing fibrosis. The severity of liver fibrosis in NASH strongly predicts long-term outcomes including liver transplantation and overall mortality [2]. Left untreated, liver fibrosis may progress to cirrhosis, conferring increased risk of hepatocellular carcinoma and liver-related mortality. Early therapeutic intervention in patients with NASH-related fibrosis may stabilize or even reverse fibrosis [3, 4]. Accurate diagnosis and staging of liver fibrosis enable risk stratification, monitoring for progression, and targeting interventions in these patients.

Histology is the current clinical standard for assessing fibrosis stage but liver biopsy is invasive, costly, and associated with non-negligible complication risk [5]. These drawbacks make histology impractical for screening patients with NAFLD, and noninvasive methods for assessing liver fibrosis are needed. To address this need, several elastography techniques have been developed for detecting and staging fibrosis noninvasively. Two-dimensional shear wave elastography (SWE, also known as sonoelastography), an advanced ultrasound-based technique, has comparable to superior accuracy for diagnosing fibrosis in NAFLD patients compared to older ultrasound-based techniques such as transient elastography (TE) and point shear wave elastography (pSWE) [6, 7]. Magnetic resonance elastography (MRE), an MR-based technique, has shown excellent performance for diagnosing and staging fibrosis in NAFLD patients [8, 9]. While comparative evidence is accumulating, the optimal selection of SWE versus MRE remains unclear in the context of NAFLD. NAFLD may pose specific challenges to noninvasive techniques due to its association with steatosis (which alters sonographic echoes and MR signals) and obesity (which increases the abdominal wall thickness leading to potentially less reliable results). Two recent studies comparing the diagnostic performance of MRE and SWE showed either significant difference at staging cirrhosis only or no difference between the two methods in cohorts where the majority had at least significant fibrosis (stage ≥ 2) [10, 11]. These studies provide important comparative data on the performance of these methods in assessing fibrosis in patient populations with relatively advanced fibrosis stage distributions. However, studies are lacking that compare the diagnostic accuracy of MRE and SWE at staging the full spectrum of liver fibrosis severity in patients with NAFLD, particularly those who might have earlier stages of fibrosis, using histopathology as the reference standard.

The purpose of this study is to compare the diagnostic performance of SWE versus MRE for staging fibrosis in a well-characterized cohort of American adults with suspected or known NAFLD using histopathology as the reference standard. Secondarily, we explored the impact of obesity and hepatic steatosis on performance.

## Materials and methods

### Study design

This is a cross-sectional study of a prospectively recruited cohort of patients with known or suspected NAFLD who underwent liver biopsy for clinical care and contemporaneous SWE and MRE for research within 180 days of liver biopsy between July 2016 and June 2019. Confounder-corrected chemical-shift-encoded (CSE)-MRI was performed as part of the MRE exam in order to estimate proton-density fat fraction (PDFF), which was used to stratify the cohort in the exploratory analyses. This study was approved by the Institutional Review Board and is compliant with the Health Insurance Portability and Accountability Act.

Study participants were recruited at the University of California, San Diego (UCSD), NAFLD Research Center. The screening process consisted of a standardized clinical evaluation which included a detailed physical examination, biochemical profiling, and an alcohol history assessment performed using the Alcohol Use Disorders Identification Test and Skinner Lifetime Drinking questionnaires. Eligible participants provided written informed consent to undergo SWE and MRE. Participants were instructed to fast for at least 8 h prior to SWE and MRE exams.

### Histologic analysis

For this research, a single experienced hepatopathologist (M.A.V., > 10 years of experience) reviewed the clinically obtained biopsy specimen and scored the histologic features using the NASH Clinical Research Network histologic scoring system [12]. Fibrosis was scored from 0 to 4, steatosis from 0 to 3, lobular inflammation from 0 to 3, and hepatocellular ballooning from 0 to 2.

Details of eligibility criteria, clinical assessments, liver biopsy protocol, and histology interpretation are available as Supplemental Methods.

### SWE exam

SWE exams were performed on a clinical ultrasound system (GE Logiq E9, GE Healthcare) provided by GE for this research through an equipment loan agreement. The ultrasound system was equipped with the transducer and software required for SWE.

One of four certified diagnostic medical sonographers (each with > 10 years of clinical experience in abdominal US exams and at least 1 year of research experience in SWE) performed SWE using a convex transducer (C1-6). Sonographers were scheduled for each exam based on availability.

For SWE, participants were imaged in the dorsal decubitus position with the right arm fully abducted to facilitate a right intercostal approach. The transducer was oriented perpendicular to the liver capsule to optimize the acoustic window. Then, SWE was activated and, once a real-time colorized stiffness map of the right liver parenchyma had stabilized during an 8–10-s breath hold at shallow expiration, the sonographer recorded the stiffness map with a button press. The sonographer then placed a circular ROI at least 1 cm below the liver capsule but no more than 8 cm from the skin surface that overlaid as much of the homogeneous color map as possible while avoiding large blood vessels, portal tracts, and rib shadowing. The mean and standard deviation of shear wave speed values within the ROI were recorded.

The above steps were repeated until 10 sequential shear wave speed (SWS) measurements were acquired per participant (out of a maximum of 20 attempts), as recommended by the manufacturer. A study was considered adequate if the IQR for the 10 measurements was less than 30% of the median (IQR/median < 0.30)[13, 14]. The entire SWE exam lasted about 10 min.

### MR exam: MRE and chemical-shift-encoded MRI

MR exams were performed using a 3-T research scanner with a 60-cm bore (GE Discovery MR750; GE Healthcare) and a 32-channel torso radiofrequency coil array. The scanner was fitted with MRE hardware and software licensed for research (Resoundant) [15, 16]. The entire MR exam including participant positioning, MRE driver placement, and MRE and CSE-MRI acquisition lasted about 20 min.

#### MRE sequence and analysis

An active acoustic driver set to the standard frequency of 60-Hz delivered vibrations via a passive pneumatic driver that was centered over the liver and secured snugly to the abdominal wall by an elastic band. A two-dimensional (2D) gradient-recalled-echo (GRE) MRE sequence modified with bipolar motion encoding gradients synchronized to the applied vibration imaged the shear wave displacement. Four 10-mm contiguous axial slices were acquired through the widest transverse section of the liver, each with a 16-s breath hold performed at relaxed end-expiration. Acquisition parameters are listed in Supplemental Methods. Using MRE reconstruction software, the MR scanner automatically processed the wave images into cross-sectional 2D shear-stiffness maps; unreliable pixels (goodness-of-fit *R*^2^ < 95%) were cross-hatched to exclude them from analysis [17].

One of two trained image analysts (each with > 1 year of experience in MRE analysis) downloaded the raw and processed MRE data for offline analysis. Using MRE analysis software (“MRE Quant”, Resoundant), the analyst manually drew free-form ROIs on portions of the right hepatic lobe on the wave images while avoiding the liver edge (outer 1 cm), major vessels, and areas of nonplanar or low amplitude wave propagation [4, 18, 19]. The ROIs were drawn on all four slices and colocalized to the shear-stiffness maps. The mean of liver stiffness in the ROIs (shear stiffness, in kilopascals) and cumulative ROI size over four slices (in pixels) were automatically reported by the software. An MRE exam was considered adequate if the total number of pixels over four slices acquired in a participant was greater than or equal to 700 pixels [20].

#### Chemical-shift-encoded MRI acquisition and analysis

A 2D multi-echo spoiled gradient-recalled-echo sequence with magnitude reconstruction was performed through the entire liver. Using a previously described custom algorithm, the MR scanner automatically processed the source images into cross-sectional PDFF maps [21–24], which were analyzed offline to calculate mean liver PDFF values. Acquisition and analysis details are described in Supplemental Methods.

### Blinding

The pathologist was blinded to imaging data. Sonographers were blinded to clinical, histological, and MRI data. MR analysts were blinded to clinical, histological, and ultrasound data.

### Statistical analyses

Statistical analysis was performed by a postdoctoral fellow (Y.N.Z., 2 years of experience) under the supervision of a biostatistician (T.W., with 25 years of experience) using “R” statistical computing software (R version 3.4.2 [2016]; R Foundation for Statistical Computing).

Sample size was based on feasibility. The target enrollment was set to ≥ 100 participants who complied with the study protocol and completed SWE and MRE.

#### Diagnostic performance

Analyses of diagnostic performance were performed in participants in whom both SWE and MRE were adequate, as defined earlier. Spearman’s correlation was used to evaluate the relationship between SWE, MRE, and fibrosis stages.

ROCs and AUCs with DeLong 95% confidence intervals (CIs) were computed for SWE and MRE for classifying each dichotomized fibrosis stage. AUCs were compared using the DeLong test for dependent ROCs. The shear wave speed cutoffs (SWE) or stiffness cutoffs (MRE) providing at least 90% sensitivity or at least 90% specificity for each dichotomization were identified. Performance parameters at those cutoffs were compared using McNemar’s test for paired proportions. The Bonferroni correction was applied to each grouped comparison of AUC, sensitivity at 90% specificity, and specificity at 90% sensitivity. A *p* value less than 0.05 (or individual *p* value < 0.05/3 = 0.017 after the Bonferroni correction) was considered statistically significant. We chose a priori not to formally compare additional performance metrics (PPV, NPV, total accuracy) to reduce the number of comparisons.

#### Exploratory analyses

To evaluate the impact of obesity and steatosis on both techniques, the above analyses were repeated separately in obese (BMI ≥ 30) and nonobese (BMI < 30) participants and in those with none-to-mild and moderate-to-severe steatosis as determined noninvasively by published PDFF cutoffs (none-to-mild: PDFF < 17.43%; moderate-to-severe: PDFF ≥ 17.43%) [21].

## Results

### Participants

**Fig. 1:**
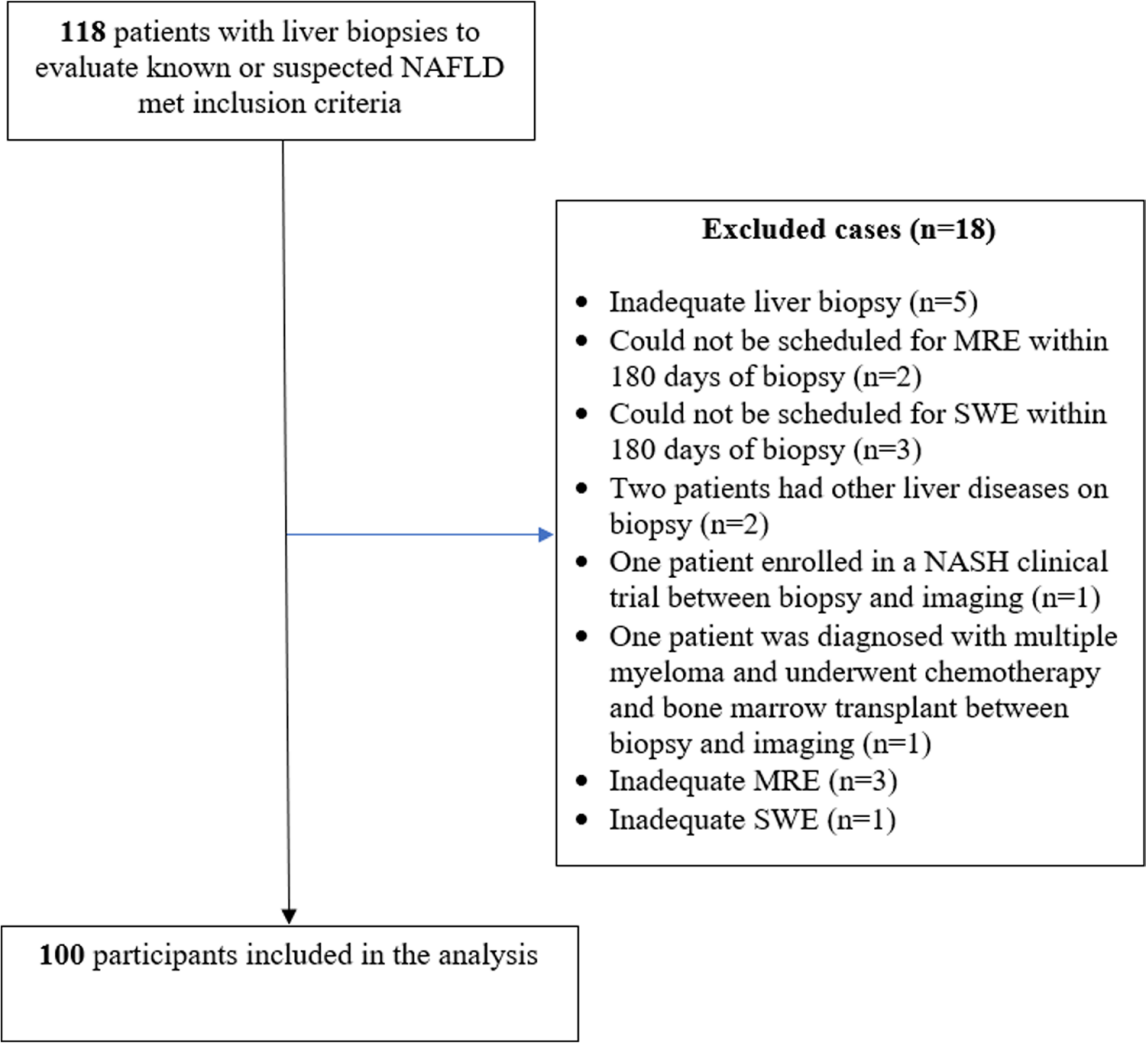
Flowchart of participant selection. *NAFLD*, nonalcoholic fatty liver disease; *NASH*, nonalcoholic steatohepatitis; *SWE*, shear wave elastography; *MRE*, magnetic resonance elastography

Between July 2016 and June 2019, 118 patients with liver biopsies to evaluate known or suspected NAFLD met inclusion criteria, of whom 18 were excluded (Fig. 1), leaving 100 participants in the final analysis.

Table 1 summarizes the baseline demographic, biochemical, histological, and imaging data for these 100 participants. Sixty-four participants (64%) were obese (BMI ≥ 30.0). The median PDFF was 14.2%. Median time intervals were 27 days between SWE and biopsy, 28 days between MRE and biopsy, and 0 days between SWE and MRE. The average (± standard deviation [SD]) of biopsy size and number of portal triads were 21.8 (± 7.3) mm and 14.5 (± 3.7), respectively. In total, 43, 36, 5, 10, and 6 had fibrosis stages 0, 1, 2, 3, and 4, respectively.

**Table 1:** Demographic, biochemical, histological, and imaging characteristics of study participants

Characteristic	Values
Demographic, anthropometric, biochemical, and imaging data (*n* = 100)	
Participant sex	
Male	46 (46%)
Female	54 (54%)
Mean age (year), mean ± SD [range]	51.8 ± 12.9 [25–78]
Mean body mass index (kg/m^2^), mean ± SD [range]	31.6 ± 4.7 [21.5–43.3]
Self-reported race	
Black or African American	3 (3%)
Asian	16 (16%)
White	55 (55%)
Other	26 (26%)
Self-reported ethnicity	
Hispanic	33 (33%)
Non-Hispanic	66 (66%)
Declined to state	1 (1%)
Biochemical profile, mean ± SD	
Aspartate aminotransferase level (U/L)	43.7 ± 18.6
Alanine aminotransferase level (U/L)	64.7 ± 36.9
Alkaline phosphatase level (IU/L)	83.2 ± 20.1
Total bilirubin (mg/dL)	0.5 ± 0.2
Albumin level (g/dL)	4.4 ± 0.7
Glucose (mg/dL)	116.0 ± 32.9
Platelet count (billion/L)	270.2 ± 71.1
Prothrombin time (s)	11.1 ± 0.7
International normalized ratio	1.0 ± 0.1
Biopsy sample, mean ± SD	
Sample length (mm)	21.8 ± 7.3
No. of portal tracts	14.5 ± 3.7
Histology, *n* (%)	
Fibrosis	
0 (no fibrosis)	43 (43%)
1 (perisinusoidal or periportal)	36 (36%)
2 (perisinusoidal and periportal)	5 (5%)
3 (bridging fibrosis)	10 (10%)
4 (cirrhosis)	6 (6%)
Steatosis	
0 (< 5% hepatocytes)	6 (6%)
1 (5–33% hepatocytes)	43 (43%)
2 (33–66% hepatocytes)	40 (40%)
3 (> 66% hepatocytes)	11 (11%)
Lobular inflammation	
0 (no foci)	7 (7%)
1 (< 2 foci per 200 × field)	68 (68%)
2 (2–4 foci per 200 × field)	19 (19%)
3 (> 4 foci per 200 × field)	6 (6%)
Ballooning	
0 (no ballooned cells)	51 (51%)
1 (few ballooned cells)	45 (45%)
2 (many ballooned cells or prominent ballooning)	4 (4%)
Imaging	
SWE, m/s, mean ± SD	1.5 ± 0.2
MRE, kPa, mean ± SD	2.6 ± 0.9
MRI-PDFF—per protocol, %, mean (median) ± SD	13.9 (14.2) ± 8.1
SWE to biopsy time interval, days, mean (median) ± SD	37.6 (27) ± 34.8
MRE to biopsy time interval, days, mean (median) ± SD	33.3 (28) ± 31.7
SWE to MRE time interval, days, mean (median) ± SD	15.3 (0) ± 26.9

*SWE* shear wave elastography, *MRE* magnetic resonance elastography, *m/s* meters per second, *kPa* kilopascals

### Diagnostic performance

**Fig. 2:**
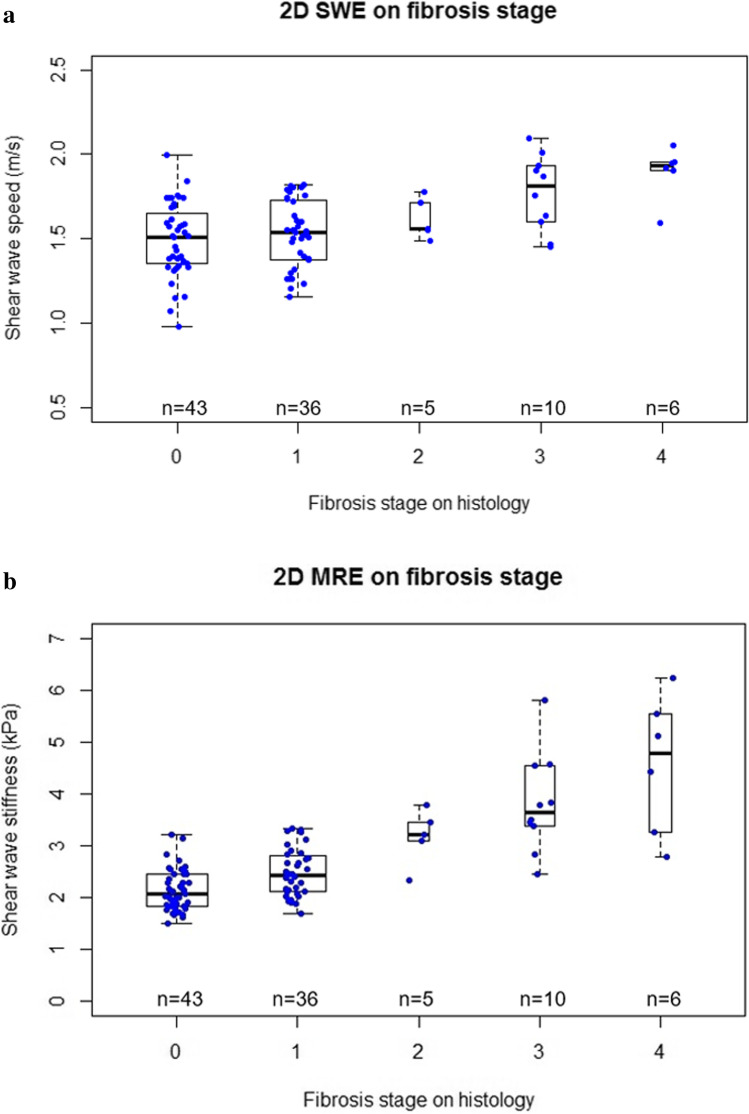
Distribution of shear wave speed measurements by shear wave elastography (**a**) and liver stiffness measurements by magnetic resonance elastography (**b**) stratified by biopsy-determined fibrosis stage (Nonalcoholic Steatohepatitis Clinical Research Network)

**Fig. 3:**
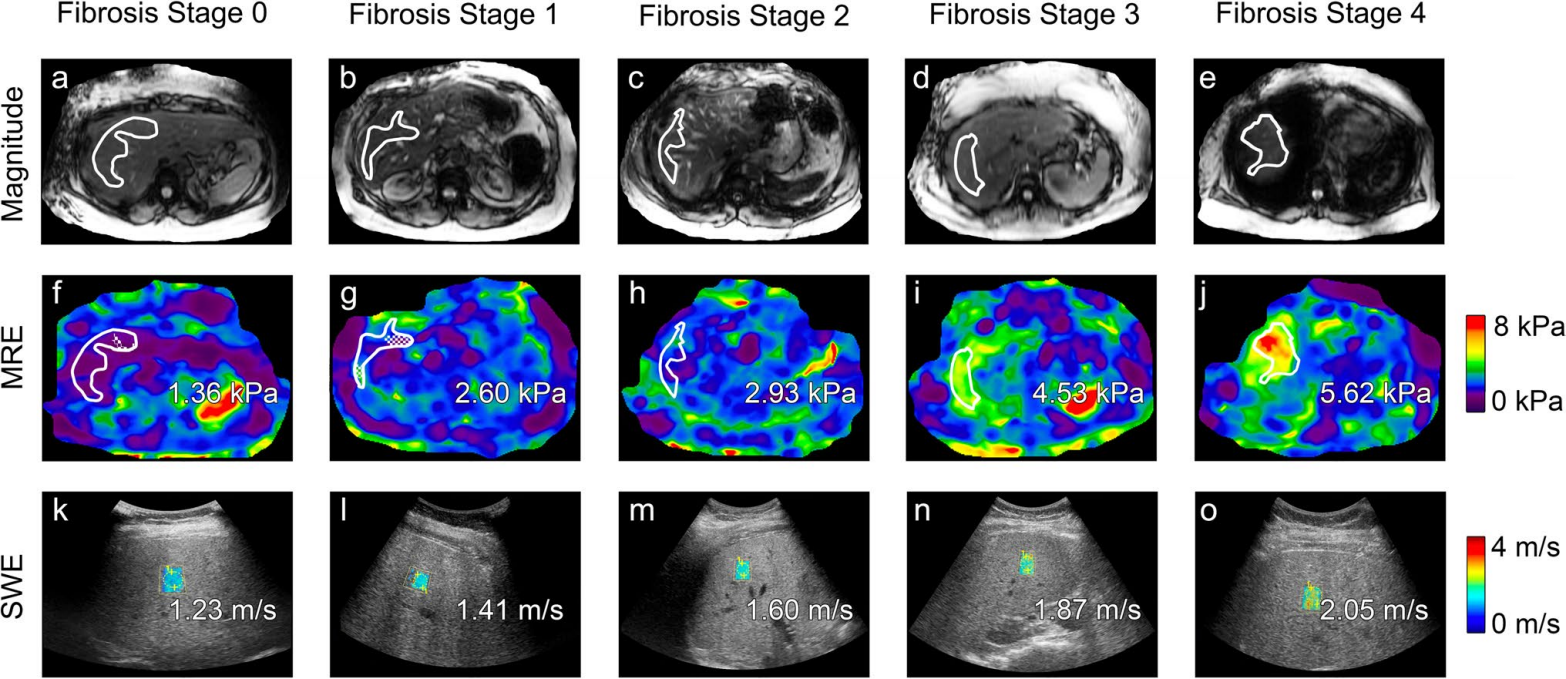
Transverse colorized MR elastograms (3-T GE 750 scanner using 2D GRE technique, top) and ultrasound-based SWE images with colorized elasticity in the ROIs (GE Logiq E9 with C1-6 transducer, bottom) demonstrate increasing shear stiffness estimates (kPa) or shear wave speed estimates (m/s) as histologically determined liver fibrosis stage (Nonalcoholic Steatohepatitis Clinical Research Network) increases in patients with nonalcoholic fatty liver disease. From left to right: stage 0 in a 46-year-old woman; stage 1 in a 56-year-old woman; stage 2 in a 44-year-old man; stage 3 in a 42-year-old woman; stage 4 in a 68-year-old woman. Magnitude of complex modulus in kPa, ROIs, and an automated confidence grid set to 95% are overlain on the MR elastograms. ROIs depicted in the MRE imaging examples for stages 0, 1, 2, 3, and 4 are 158 cm^2^, 63 cm^2^, 80 cm^2^, 90 cm^2^, and 126 cm^2^, respectively. Shear wave speed estimates are overlain on the SWE images. ROIs depicted in the SWE imaging examples for stages 0, 1, 2, 3, and 4 are 1.2 cm^2^, 0.7 cm^2^, 0.9 cm^2^, 1.1 cm^2^, and 0.9 cm^2^, respectively

Mean shear wave speed and stiffness values are shown in Fig. 2. Mean shear wave speed and stiffness values increased monotonically with fibrosis stages (Spearman’s correlation coefficient for shear wave speed values and fibrosis stages is 0.392 (*p* < 0.01), and for stiffness values and fibrosis stages is 0.654 (*p* < 0.01). Representative SWE and MRE images are shown in Fig. 3. The mean (± SD) area of the captured SWE ROIs was 1.19 (± 0.39) cm^2^, and the mean (± SD) cumulative ROI size over 4 slices of MRE for each participant was 3350 (± 1498) pixels (469 cm^2^ ± 210 cm^2^).

The AUCs of MRE were significantly higher than those of SWE for diagnosing any fibrosis (0.81 [95% CI: 0.72–0.89] vs. 0.65 [95% CI: 0.54–0.76], *p* = 0.005) and stage ≥ 2 fibrosis (0.94 [95% CI: 0.89–1.00] vs. 0.81 [95% CI: 0.71–0.91], *p* = 0.009). The AUC point estimates of MRE were nominally higher than those of SWE for stage ≥ 3 and stage = 4 fibrosis, but the differences were not significant (Table 2).

**Table 2:** AUCs and AUC comparisons for SWE and MRE by dichotomized fibrosis stage

Method	Fibrosis stage 0 vs. 1–4	Fibrosis stage 0–1 vs. 2–4	Fibrosis stage 0–2 vs. 3–4	Fibrosis stage 0–3 vs. 4
All participants (*n* = 100)				
	Stage 0 = 43; stage 1–4 = 57	Stage 0–1 = 79; stage 2–4 = 21	Stage 0–2 = 84; stage 3–4 = 16	Stage 0–3 = 94; stage 4 = 6
SWE	0.65 (0.54–0.76)	0.81 (0.71–0.91)	0.85 (0.74–0.96)	0.91 (0.79–1.00)
MRE	0.81 (0.72–0.89)	0.94 (0.89–1.00)	0.95 (0.89–1.00)	0.92 (0.83–1.00)
* p*^a^	0.005*	0.009*	0.053	0.720
Subset of obese participants (*n* = 64)				
	Stage 0 = 24; stage 1–4 = 40	Stage 0–1 = 48; stage 2–4 = 16	Stage 0–2 = 51; stage 3–4 = 13	Stage 0–3 = 59; stage 4 = 5
SWE	0.65 (0.51–0.79)	0.81 (0.69–0.92)	0.83 (0.70–0.96)	0.90 (0.75–1.00)
MRE	0.84 (0.74–0.94)	0.92 (0.85–1.00)	0.93 (0.87–1.00)	0.90 (0.78–1.00)
* p*^a^	0.008*	0.056	0.093	0.972
Subset of nonobese participants (*n* = 36)				
	Stage 0 = 20; stage 1–4 = 16	Stage 0–1 = 31; stage 2–4 = 5	Stage 0–2 = 33; stage 3–4 = 3	Stage 0–3 = 35; stage 4 = 1
SWE	0.66 (0.48–0.85)	0.85 (0.62–1.00)	0.99 (0.96–1.00)	NA
MRE	0.75 (0.58–0.92)	0.99 (0.98–1.00)	1.00 (1.00–1.00)	NA
* p*^a^	0.354	0.214	0.480	NA
Subset of participants with moderate-to-severe steatosis (PDFF^a^ ≥ 17.43%) (*n* = 32)				
	Stage 0 = 10; stage 1–4 = 22	Stage 0–1 = 27; stage 2–4 = 5	Stage 0–2 = 29; stage 3–4 = 3	Stage 0–3 = 31; stage 4 = 1
SWE	0.59 (0.34–0.83)	0.77 (0.57–0.97)	0.74 (0.41–1.00)	NA
MRE	0.83 (0.68–0.98)	0.81 (0.63–1.00)	0.82 (0.60–1.00)	NA
* p*^a^	0.024*	0.655	0.424	NA
Subset of participants with none-to-mild steatosis (PDFF^a^ < 17.43%) (*n* = 68)				
	Stage 0 = 33; stage 1–4 = 35	Stage 0–1 = 52; stage 2–4 = 16	Stage 0–2 = 55; stage 3–4 = 13	Stage 0–3 = 63; stage 4 = 5
SWE	0.72 (0.59–0.84)	0.82 (0.70–0.95)	0.88 (0.76–0.99)	0.96 (0.92–1.00)
MRE	0.82 (0.72–0.92)	0.98 (0.96–1.00)	0.98 (0.95–1.00)	0.96 (0.90–1.00)
* p*^a^	0.127	0.009*	0.075	0.883

*AUC* area under the receiver operating characteristic curve, *SWE* shear wave elastography, *MRE* magnetic resonance elastography, *PDFF* proton-density fat fraction; 95% confidence interval in parenthesis

^a^Mean proton-density fat fraction (%) of 9 liver segments

^*^p value, as calculated by DeLong’s test to compare the AUCs of MRE and SWE. Using Bonferroni correction, individual *p* value < 0.05/3 (for grouped AUC, sensitivity, and specificity) is considered significant (asterisk)

Cutoffs and performance parameters for the classification of dichotomized fibrosis stages given predefined sensitivity or specificity ≥ 90% are summarized in Tables 3 and 4.

**Table 3:** Diagnostic performance of SWE and MRE at classifying dichotomized fibrosis stages for predefined sensitivity ≥ 90%

Fibrosis stage	*N* positive	*N* negative	Method	Cutoff	Sensitivity	Specificity	PPV	NPV	Accuracy
Stage 1–4 vs. 0	57	43	MRE	2.01 kPa	0.912	0.488*	0.703	0.808	0.730
			SWE	1.27 m/s	0.912	0.116	0.578	0.500	0.570
Stage 2–4 vs. 0–1	21	79	MRE	2.77 kPa	0.905	0.848*	0.613	0.971	0.860
			SWE	1.49 m/s	0.905	0.430	0.297	0.944	0.530
Stage 3–4 vs. 0–2	16	84	MRE	2.77 kPa	0.938	0.810*	0.484	0.986	0.830
			SWE	1.46 m/s	0.938	0.393	0.227	0.971	0.480
Stage 4 vs. 0–3	6	94	MRE	2.77 kPa	1.000	0.734	0.194	1.000	0.750
			SWE	1.59 m/s	1.000	0.617	0.143	1.000	0.640

*MRE* magnetic resonance elastography, *SWE* shear wave elastography, *PPV* positive predictive value, *NPV* negative predictive value; *kPa* kilopascals, unit for shear stiffness as measured by MRE; *m/s* meters per second, unit for shear wave speed as measured by SWE

^*^Specificity of MRE is significantly higher than that of SWE based on two-tailed McNemar’s test, *p* < 0.001. Using Bonferroni correction, individual *p* value < 0.05/3 (for grouped AUC, sensitivity, and specificity) is considered significant

**Table 4:** Diagnostic performance of SWE and MRE at classifying dichotomized fibrosis stages for predefined specificity ≥ 90%

Fibrosis stage	*N* positive	*N* negative	Method	Cutoff	Sensitivity	Specificity	PPV	NPV	Accuracy
Stage 1–4 vs. 0	57	43	MRE	2.60 kPa	0.579*	0.907	0.892	0.619	0.720
			SWE	1.75 m/s	0.333	0.907	0.826	0.506	0.580
Stage 2–4 vs. 0–1	21	79	MRE	3.06 kPa	0.810*	0.911	0.708	0.947	0.890
			SWE	1.79 m/s	0.476	0.911	0.588	0.867	0.720
Stage 3–4 vs. 0–2	16	84	MRE	3.17 kPa	0.813	0.905	0.619	0.962	0.890
			SWE	1.78 m/s	0.625	0.905	0.556	0.927	0.860
Stage 4 vs. 0–3	6	94	MRE	3.42 kPa	0.667	0.904	0.308	0.977	0.890
			SWE	1.81 m/s	0.833	0.904	0.357	0.988	0.900

*MRE* magnetic resonance elastography, *SWE* shear wave elastography, *PPV* positive predictive value, *NPV* negative predictive value; *kPa* kilopascals, unit for shear stiffness as measured by MRE; *m/s* meters per second, unit for shear wave speed as measured by SWE

^*^Sensitivity of MRE is significantly higher than that of SWE based on two-tailed McNemar’s test, *p* ≤ 0.01. Using Bonferroni correction, individual *p* value < 0.05/3 (for grouped AUC, sensitivity, and specificity) is considered significant

At sensitivity of at least 90%, the SWE cutoffs were 1.27, 1.49, 1.46, and 1.59 m/s for stage ≥ 1 fibrosis, stage ≥ 2 fibrosis, stage ≥ 3 fibrosis, and stage 4 fibrosis, respectively; the MRE cutoffs were 2.01, 2.77, 2.77, and 2.77 kPa, respectively. MRE had higher specificity than SWE for all stages of fibrosis, and the difference was significant for stage ≥ 1, ≥ 2, and ≥ 3 (*p* < 0.001). The point estimate for PPV was higher for MRE than for SWE for all stages of fibrosis among this particular cohort, though formal statistical comparisons were not performed.

At specificity of at least 90%, the SWE cutoffs were 1.75, 1.79, 1.78, and 1.81 m/s for stage ≥ 1 fibrosis, stage ≥ 2 fibrosis, stage ≥ 3 fibrosis, and stage 4 fibrosis, respectively; the MRE cutoffs were 2.60, 3.06, 3.17, and 3.42 kPa, respectively. MRE had higher sensitivity than SWE for all stages of fibrosis, and the difference was significant for fibrosis stages ≥ 1 and ≥ 2 (*p* ≤ 0.01). The point estimate for NPV was higher for MRE than for SWE for all stages of fibrosis except cirrhosis (stage 4) among this particular cohort, though formal statistical comparisons were not performed.

### Exploratory analyses

In stratified analysis of obese (*n* = 64) and nonobese (*n* = 36) participants, MRE was superior to SWE at diagnosing stage ≥ 1 fibrosis among obese participants (*p* = 0.008). In stratified analysis of participants with none-to-mild steatosis using PDFF cutoff < 17.43% (*n* = 68) and moderate-to-severe steatosis using PDFF cutoff ≥ 17.43% (*n* = 32), MRE was superior to SWE at diagnosing stage ≥ 2 fibrosis among participants with none-to-mild steatosis (*p* = 0.009), and at diagnosing stage ≥ 1 fibrosis among participants with moderate-to-severe steatosis (*p* = 0.024).

## Discussion

Noninvasive imaging methods for estimating fibrosis in NAFLD patients have been suggested both for initial detection and staging and for longitudinal monitoring, a scenario in which invasive tests like biopsy are not feasible. Patients with NAFLD pose several challenges (e.g., obesity, steatosis) that may impact imaging study performance. Hence, the optimal test or combination of tests has yet to be defined. Our study aimed to compare MRE and SWE against histological reference standard in a NAFLD population. While MRE was significantly more accurate than SWE for diagnosing lower stages of fibrosis (stage ≥ 1 and ≥ 2), the two techniques did not differ significantly at higher stages of fibrosis (stage ≥ 3 and = 4). In exploratory analyses, MRE also showed a trend towards better performance than SWE in all participant subgroups regardless of the presence of obesity or the severity of steatosis, though the differences between subgroups were sometimes significant only in the lower fibrosis stages.

Our study is the first to detect a significant difference in performance between SWE and MRE at diagnosing lower stages of fibrosis. A previous study by Furlan et al.on American adults with NAFLD examined the diagnostic performance of SWE and MRE at detecting significant fibrosis (stage ≥ 2) and advanced fibrosis (stage ≥ 3) and did not find a statistically significant difference, while a recent study by Imajo et al. on Japanese adults with NAFLD examined the diagnostic performance of SWE and MRE at detecting the full spectrum of fibrosis and found that MRE offered superior performance at staging cirrhosis only [10, 11]. The small number of participants in both studies who had no liver fibrosis (1 in Furlan et al. and 9 in Imajo et al. had no liver fibrosis) may have limited their statistical power for comparing the diagnostic performance of SWE and MRE for detecting any fibrosis. Similarly, the relatively small number of participants in both studies who had mild disease (fibrosis stage < 2) (18 in Furlan et al. and 59 in Imajo et al., compared to 79 in our study) may have reduced the power to detect differences in performance at diagnosing earlier stages of fibrosis. At advanced fibrosis (stage ≥ 3), we—like Furlan et al.and Imajo et al.—found that there is no statistically significant difference in performance between SWE and MRE. Conversely, the small number of participants in our study with cirrhosis (6 out of 100) likely reduced our power to detect differences in performance for diagnosing cirrhosis, and may explain why our results do not replicate the finding by Imajo et al. that MRE is superior to SWE for diagnosing cirrhosis. Despite the small number of participants with cirrhosis, our overall cohort was comparatively large, which allowed for exploratory analysis of NAFLD patients stratified by fibrosis severity and obesity, two potential confounders for noninvasive techniques.

Compared to published studies on the diagnostic performance of SWE for fibrosis staging in NAFLD patients, we found lower diagnostic accuracy as assessed with AUCs [7, 10, 25–27]. Differences in stage distribution may account in part for the discrepancy. A majority of participants (58.3% to 78.4%) in published studies had stage ≥ 2 fibrosis compared to a minority (21%) in our cohort. The higher proportion of patients with more severe fibrosis in published studies is expected to increase the observed AUC, since greater separation between shear wave speed or shear stiffness values are observed at higher fibrosis stages [28]. Compared to study cohorts that skew towards the more severe spectrum of liver fibrosis, our results may be most applicable to the outpatient NAFLD hepatology clinic from which we enrolled our participants.

The diagnostic performance of MRE across all dichotomized fibrosis stages in our study was consistent with prior studies on NAFLD patients and overweight-to-obese patients, which included patients with similar fibrosis stage distribution [8, 9, 29]. We intentionally reported two sets of cutoff values for MRE and SWE—one set that would yield at least 90% sensitivity and one set that would yield at least 90% specificity—for each dichotomized fibrosis stage instead of the Youden index. While the Youden index maximizes the combination of sensitivity and specificity for a particular test, it is not as helpful in informing clinical application and interpretation. For instance, recent guidelines from the American Association for the Study of Liver Diseases (AASLD) suggest the use of noninvasive tests to detect patients with high likelihood of advanced stage fibrosis—i.e., those patients who may have the greatest benefit-to-risk ratio for biopsy [30]. This context of use requires high sensitivity and NPV to rule out fibrosis in order to appropriately direct biopsy to those at high risk. For this purpose, SWE and MRE did not differ in performance: SWE can accurately exclude stage ≥ 3 fibrosis with sensitivity of 94–100% and NPV of 97–100% while MRE can do so with sensitivity of 94–100% and NPV of 99–100%.

As opposed to ruling out disease, ruling in disease requires high specificity and PPV. Although we identified high-specificity (≥ 90%) cutoffs, our cohort was assembled from an outpatient setting, where the pre-test probability of advanced fibrosis tends to be low. In this situation, despite applying high-specificity cutoffs, the PPVs for ruling in advanced fibrosis (62% PPV for MRE, 56% PPV for SWE) are not sufficient to avoid biopsy altogether. Our results are consistent with those reported by Loomba et al., where an MRE stiffness cutoff of 3.63 kPa yielded a specific result (91%) and a high NPV of 97% for excluding stage ≥ 3 fibrosis in NAFLD patients, but a PPV of only 68% for ruling it in [31]. Furlan et al.reported similar MRE stiffness cutoff of 3.4 kPa for excluding stage ≥ 3 fibrosis with a specificity of 91.7%, but a lower NPV of 91.7% and a much higher PPV of 87% compared to our results [10]. The higher prevalence of stage ≥ 3 fibrosis in Furlan et al. compared to this study (39% versus 16%) contributed at least in part to the differences in reported NPV and PPV. Thus, if confirmation of advanced fibrosis is desired, then further evaluation possibly including a liver biopsy may be needed. Combining noninvasive tests with clinical decision support tools such as the NAFLD fibrosis score or the FIB-4 test might also improve the PPV [32, 33].

Our study has several limitations. First, the small sample sizes of obese and nonobese subsets as well as the nonuniform stratification of steatosis severity by PDFF cutoff values limited our assessment of obesity and steatosis and their confounding effects on SWE and MRE. Future studies are needed to verify our preliminary finding from the exploratory analyses that MRE is superior to SWE regardless of body habitus and steatosis severity. Also, the distribution of liver fibrosis in our cohort is skewed towards the milder end of the spectrum. Although this may increase the applicability of our results to common clinical contexts such as fibrosis screening, the relatively low number of participants with fibrosis stage ≥ 2 compared to fibrosis stage 0–1 limits discrimination between adjacent advanced fibrosis stages. For instance, for a predefined sensitivity ≥ 90%, MRE cutoff is the same for fibrosis stages 2, 3, and 4 (2.77 kPa) while SWE cutoff for fibrosis stage 3 is lower than for fibrosis stage 2. For the purposes of comparing SWE and MRE, fibrosis distribution affects both techniques equally and would not introduce a bias in favor of one method. Second, this study was conducted using US and MRI systems from a single manufacturer at a single subspecialty center focused on NAFLD research, which may limit the generalizability of its results to other settings such as community centers or sites with systems from other vendors. Third, as technology advances rapidly, it is possible that newer technologies would have provided more accurate performance. For SWE, this might include the use of software that provides real-time feedback on the quality of shear wave propagation and the use of time-harmonic elastography techniques in obese patients. For MRE, this might include a spin-echo echo planar imaging sequence rather than a GRE sequence and/or the use of thin flexible blanket-like torso phased array coils. Finally, we did not test the longitudinal reproducibility of the two modalities, a factor that would be important for determining the best test for monitoring treatment response. The comparative performance of SWE and MRE for longitudinal monitoring remains a gap in knowledge for which future research is needed.

In conclusion, this prospective study provided direct comparison of SWE versus MRE for staging fibrosis in a cohort of participants with known or suspected NAFLD and clinically indicated liver biopsy. We showed that in patients in whom both methods are adequate, MRE had significantly higher accuracy than SWE for diagnosing earlier (≥ 1 and ≥ 2) fibrosis stages. For purposes of directing biopsy to detect advanced fibrosis, SWE and MRE performed equally well, both demonstrating high NPV for excluding disease. Future studies that aim to evaluate the relative reproducibility of these modalities for longitudinal monitoring and the cost-effectiveness of various diagnostic approaches using combinations of SWE, MRE, biopsy, and clinical decision support will further inform optimal usage of both methods for clinical care and clinical trials.

## Supplementary Information

Below is the link to the electronic supplementary material.

Supplementary file1 (DOCX 1014 KB)

## Abbreviations

2D: Two-dimensional
AASLD: American Association for the Study of Liver Diseases
BMI: Body mass index
CI: Confidence interval
CSE: Confounder-corrected chemical-shift-encoded
GRE: Gradient-recalled-echo
IQR: Interquartile range
MRE: Magnetic resonance elastography
NAFLD: Nonalcoholic fatty liver disease
NASH: Nonalcoholic steatohepatitis
PDFF: Proton-density fat fraction
pSWE: Point shear wave elastography
SWE: Ultrasound shear wave elastography
SWS: Shear wave speed
TE: Transient elastography

## Declarations

### Guarantor

The scientific guarantor of this publication is Dr. Claude B. Sirlin.

### Statistics and biometry

Tanya Wolfson, MA, kindly provided statistical advice for this manuscript and is one of the authors.

### Methodology

•Cross-sectional study

## References

[1] Younossi ZM, Koenig AB, Abdelatif D (2016). Global epidemiology of nonalcoholic fatty liver disease-meta-analytic assessment of prevalence, incidence, and outcomes. Hepatology.

[2] Hagström H, Nasr P, Ekstedt M (2017). Fibrosis stage but not NASH predicts mortality and time to development of severe liver disease in biopsy-proven NAFLD. J Hepatol.

[3] Musso G, Cassader M, Paschetta E, Gambino R (2017). Thiazolidinediones and advanced liver fibrosis in nonalcoholic steatohepatitis. JAMA Intern Med.

[4] Loomba R, Lawitz E, Mantry PS (2018). The ASK1 inhibitor selonsertib in patients with nonalcoholic steatohepatitis: a randomized, phase 2 trial. Hepatology.

[5] Tapper EB, Lok AS-F (2017). Use of liver imaging and biopsy in clinical practice. N Engl J Med.

[6] Lee MS, Bae JM, Joo SK et al (2017) Prospective comparison among transient elastography, supersonic shear imaging, and ARFI imaging for predicting fibrosis in nonalcoholic fatty liver disease. PLoS One 1–17. 10.1371/journal.pone.0188321 10.1371/journal.pone.0188321 PMC5703509 29176844

[7] Cassinotto C, Boursier J, de Lédinghen V (2016). Liver stiffness in nonalcoholic fatty liver disease: a comparison of supersonic shear imaging, FibroScan, and ARFI with liver biopsy. Hepatology.

[8] Cui J, Heba E, Hernandez C (2016). Magnetic resonance elastography is superior to acoustic radiation force impulse for the diagnosis of fibrosis in patients with biopsy-proven nonalcoholic fatty liver disease: a prospective study. Hepatology.

[9] Singh S, Venkatesh SK, Loomba R (2016). Magnetic resonance elastography for staging liver fibrosis in non-alcoholic fatty liver disease: a diagnostic accuracy systematic review and individual participant data pooled analysis. Eur Radiol.

[10] Furlan A, Tublin ME, Yu L et al (2020) Comparison of 2D shear wave elastography, transient elastography, and MR elastography for the diagnosis of fibrosis in patients with nonalcoholic fatty liver disease. AJR Am J Roentgenol 214:W20–W26. 10.2214/AJR.19.21267 10.2214/AJR.19.21267 31714842

[11] Imajo K, Honda Y, Kobayashi T (2020). Direct comparison of US and MR elastography for staging liver fibrosis in patients with nonalcoholic fatty liver disease. Clin Gastroenterol Hepatol.

[12] Kleiner DE, Brunt EM, Van Natta M (2005). Design and validation of a histological scoring system for nonalcoholic fatty liver disease. Hepatology.

[13] Dietrich C, Bamber J, Berzigotti A et al (2017) EFSUMB guidelines and recommendations on the clinical use of liver ultrasound elastography, update 2017 (long version). Ultraschall Med 38:e16–e47. 10.1055/s-0043-103952 10.1055/s-0043-103952 28407655

[14] Committee USB (2017) QIBA SWS profile checklist for fibrosis 2017. In: Ultrasound SWS Biomark. Comm. http://qibawiki.rsna.org/index.php/Ultrasound_SWS_Biomarker_Ctte. Accessed 8 Nov 2018

[15] Wagner M, Corcuera-Solano I, Lo G (2017). Technical failure of MR elastography examinations of the liver: experience from a large single-center study. Radiology.

[16] Yin M, Talwalkar JA, Glaser KJ (2007). Assessment of hepatic fibrosis with magnetic resonance elastography. Clin Gastroenterol Hepatol.

[17] Dzyubak B, Glaser K, Yin M (2013). Automated liver stiffness measurements with magnetic resonance elastography. J Magn Reson Imaging.

[18] Yin M, Glaser KJ, Talwalkar JA (2016). Hepatic MR elastography: clinical performance in a series of 1377 consecutive examinations. Radiology.

[19] Park CC, Nguyen P, Hernandez C (2017). Magnetic resonance elastography vs transient elastography in detection of fibrosis and noninvasive measurement of steatosis in patients with biopsy-proven nonalcoholic fatty liver disease. Gastroenterology.

[20] Jayakumar S, Middleton MS, Lawitz EJ (2019). Longitudinal correlations between MRE, MRI-PDFF, and liver histology in patients with non-alcoholic steatohepatitis: Analysis of data from a phase II trial of selonsertib. J Hepatol.

[21] Tang A, Desai A, Hamilton G (2015). Accuracy of MR imaging–estimated proton density fat fraction for classification of dichotomized histologic steatosis grades in nonalcoholic fatty liver disease. Radiology.

[22] Tang A, Tan J, Sun M (2013). Nonalcoholic fatty liver disease: MR imaging of liver proton density fat fraction to assess hepatic steatosis. Radiology.

[23] Loomba R, Sirlin CB, Ang B (2015). Ezetimibe for the treatment of nonalcoholic steatohepatitis: assessment by novel magnetic resonance imaging and magnetic resonance elastography in a randomized trial (MOZART trial). Hepatology.

[24] Middleton MS, Heba ER, Hooker CA (2017). Agreement between magnetic resonance imaging proton density fat fraction measurements and pathologist-assigned steatosis grades of liver biopsies from adults with nonalcoholic steatohepatitis. Gastroenterology.

[25] Xiao G, Zhu S, Xiao X (2017). Comparison of laboratory tests, ultrasound, or magnetic resonance elastography to detect fibrosis in patients with nonalcoholic fatty liver disease: a meta-analysis. Hepatology.

[26] Herrmann E, de Lédinghen V, Cassinotto C (2018). Assessment of biopsy-proven liver fibrosis by two-dimensional shear wave elastography: an individual patient data-based meta-analysis. Hepatology.

[27] Yoon JH, Lee JM, Joo I (2014). Hepatic fibrosis: prospective comparison of MR elastography and US shear-wave elastography for evaluation. Radiology.

[28] Calvaruso V, Burroughs AK, Standish R (2009). Computer-assisted image analysis of liver collagen: relationship to Ishak scoring and hepatic venous pressure gradient. Hepatology.

[29] Chen J, Yin M, Talwalkar JA (2017). Diagnostic performance of MR elastography and vibration-controlled transient elastography in the detection of hepatic fibrosis in patients with severe to morbid obesity. Radiology.

[30] Chalasani N, Younossi Z, Lavine JE (2018). The diagnosis and management of nonalcoholic fatty liver disease: practice guidance from the American Association for the Study of Liver Diseases. Hepatology.

[31] Loomba R, Wolfson T, Ang B (2014). Magnetic resonance elastography predicts advanced fibrosis in patients with nonalcoholic fatty liver disease: a prospective study. Hepatology.

[32] Jung J, Loomba RR, Imajo K (2020). MRE combined with FIB-4 (MEFIB) index in detection of candidates for pharmacological treatment of NASH-related fibrosis. Gut.

[33] Angulo P, Hui JM, Marchesini G (2007). The NAFLD fibrosis score: a noninvasive system that identifies liver fibrosis in patients with NAFLD. Hepatology.

