# Supplementary material for: Comparative diagnostic performance of ultrasound shear wave elastography and magnetic resonance elastography for classifying fibrosis stage in adults with biopsy-proven nonalcoholic fatty liver disease
Source: Eur Radiol. 2021 Dec 2;32(4):2457–69. doi: 10.1007/s00330-021-08369-9 (PMC8921157; doi:10.1007/s00330-021-08369-9)
Supplement: Supplementary file 1 — Supplementary file1 (DOCX 1014 KB) [file 330_2021_8369_MOESM1_ESM.docx]

**Supplemental Methods**

**Eligibility**

Inclusion: All of the following: age ≥ 18 years; prior or anticipated clinical liver biopsy to evaluate known or suspected NAFLD; willingness and ability to undergo both SWE and MRE.

Exclusion: Any of the following: clinical, laboratory, or histologic evidence of liver disease other than NAFLD; excessive alcohol use within 2 years of recruitment (defined as ≥ 14 drinks/week for men and ≥ 7 drinks/week for women); pregnancy or trying to become pregnant; use of steatogenic or hepatotoxic medications within 6 months of recruitment; inability to present for SWE and MRE within 180 days of liver biopsy; inadequate liver biopsy (defined as sample length < 10 mm with < 3 portal tracts)[1], inadequate SWE (IQR for the 10 measurements was equal to or more than 30% of the median (IQR/median ≥ 0.30), inadequate MRE (defined as the total number of pixels over four slices acquired in a participant being less than 700 pixels) [2, 3]

**Research Visit**

All participants underwent a standardized clinical evaluation prior to scheduling ultrasound or MR exams. Study coordinators recorded age, sex, self-reported race and ethnicity, height, weight, body mass index (BMI), and vital signs under the supervision of the hepatologist (R.L., > 10 years' experience). Fasting lab results entered into the Electronic Health Record within three months of the imaging exams were recorded. Current and prior alcohol use were assessed using the Alcohol Use Disorders Identification Test (AUDIT) and the Skinner Lifetime Drinking questionnaire. Secondary causes of hepatic steatosis and other liver diseases were excluded using history, biochemical tests, hepatitis serologies, and histology findings.

**Liver Biopsy**

Hepatologists or interventional radiologists performed nontargeted percutaneous biopsies of the right or left liver lobe using an intercostal approach in a peripheral location with a 16- or 18-gauge needle for clinical care, in accordance with institutional and AASLD guidelines which do not require image guidance if adequate tissue samples can be obtained [4]. It is standard practice at our institution to acquire at least one 2-cm biopsy as determined by visual inspection. Liver biopsy specimens were fixed in formalin, embedded in paraffin, and stained with hematoxylin-eosin and Masson trichrome.

**Histologic Analysis**

For this research, a single experienced hepatopathologist (R.L., > 10 years of experience) reviewed the clinically obtained biopsy specimen and recorded the overall length and number of portal tracts visualized. Histologic features were scored using the NASH Clinical Research Network histologic scoring system [5]. Fibrosis was scored from 0 to 4, steatosis from 0 to 3, lobular inflammation from 0 to 3, and hepatocellular ballooning from 0 to 2. Histological features of liver disease other than NAFLD were recorded.

**Chemical-shift-encoded MRI acquisition and analysis**

The acquisition was performed in one or two ~20s breath holds. T1 bias was minimized by using a low flip angle of 10° with a long repetition time of >150 ms. To correct for R2* signal decay and permit quantification of fat-water signal oscillation, six echoes were obtained per repetition time (TR) at nominally out-of-phase and in-phase echo times (TEs). Parametric PDFF maps were generated by applying a fitting algorithm pixel by pixel to the magnitude source images [6] that took into account T2* decay by assuming exponential decay and the spectral complexity of fat by incorporating a multipeak fat spectrum [7].

One of two trained image analysts (E.Z.Y. and A.S.B., each with > 1 year of experience) loaded source images and PDFF maps into OsiriX software version 7.0.3 (OsiriX Foundation) and manually placed a 1-cm radius circular ROI in the center of each Couinaud liver segment on the fifth-echo source image while avoiding liver boundaries, artifacts, major vessels, and bile ducts. The ROIs were colocalized to the PDFF maps, and the mean PDFF value was reported for each segment. Whole-liver PDFF value was calculated as the mean of these nine segments.

An imaging example of PDFF analysis is shown here:


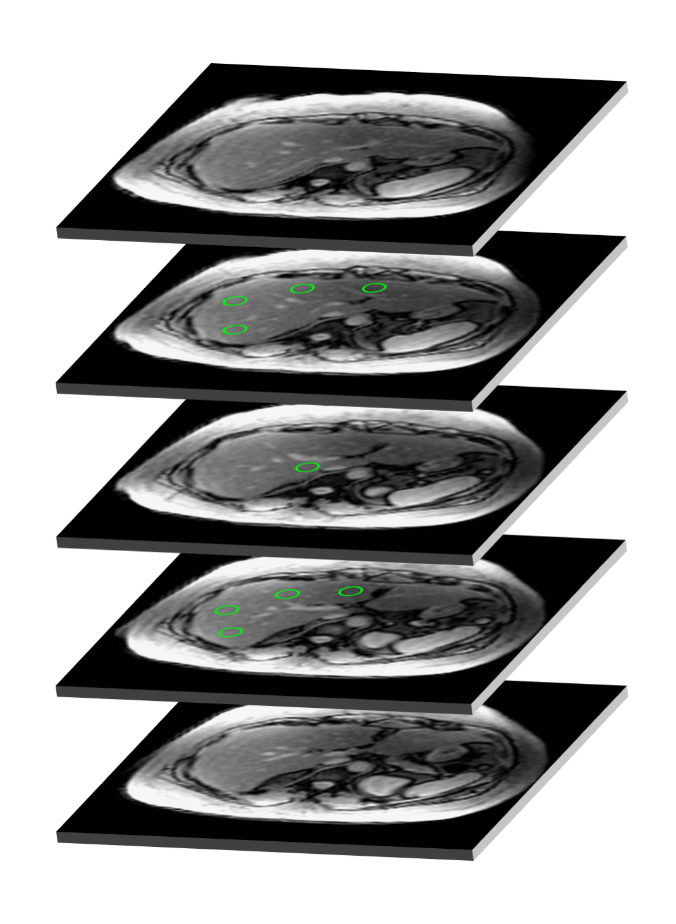


The liver is shown on the source images. The 9 regions of interest (ROIs), each 1-cm in radius and placed in the center of a Couinaud liver segment, are shown on the fifth-echo source image. The slices above and below the slices with ROIs are also shown.

**Parameters for MR Techniques**

| Acquisition Parameters | 2D GRE MRE | MRI-PDFF |
| --- | --- | --- |
| TR (ms) | 50 | ≥ 150 |
| TE (ms) | 20.2 | 1.15, 2.3, 3.45, 4.6, 5.75, 6.9 |
| FA (degrees) | 30 | 10 |
| Slice thickness (mm) | 10 | 8 |
| Number of slices | 4 | 17-33 |
| Inter-slice gap (mm) | 10 | 8 |
| Matrix | 256 × 64 | 224 × 128 (160) |
| FOV (cm) | 38-48 × 38-48 | 38-44 × 38-44 |
| BW (kHz) | ± 31.25 | ± 142 |
| Number of averages | 1 | 1 |
| Parallel imaging acceleration factor | 2 | 1.25 |

*2D* two-dimensional; *BW* Bandwidth; *FA* Flip angle; *FOV* Field of view; *GRE* gradient-recalled echo; *MRE* magnetic resonance elastography; *MRI-PDFF* magnetic resonance imaging-proton density fat fraction; *TR* repetition time; *TE* echo time

Example of MRE analysis


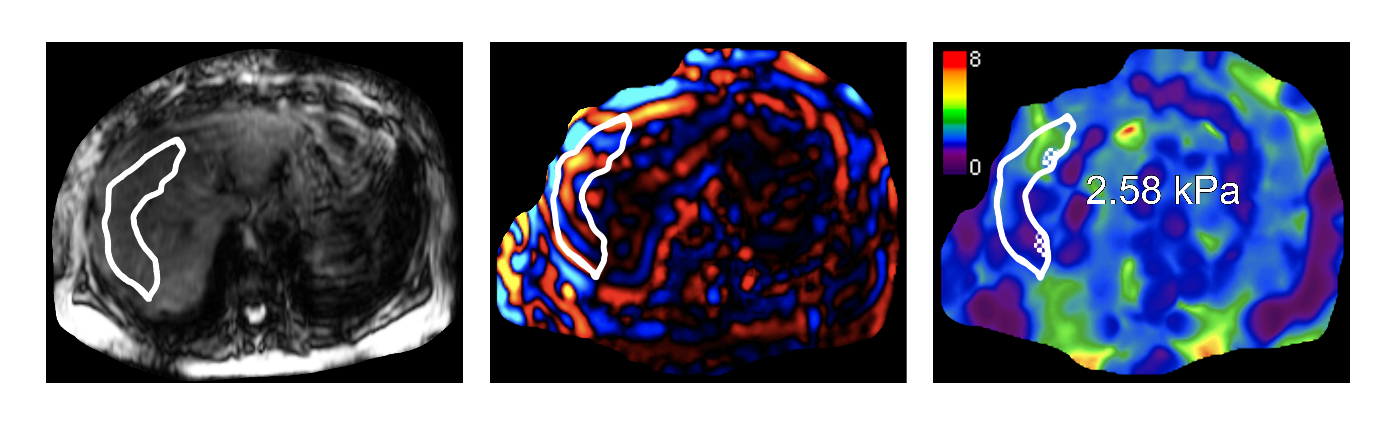


The above figure shows MR elastography performed on a 3T GE 750 scanner using a two-dimensional gradient-recalled echo (GRE) sequence. Magnitude image (*left*), transverse colorized wave image (*center*), and transverse colorized elastograms with average stiffness estimate within the region-of-interest (ROI)(*right*) are displayed. Using magnitude images as reference, the analyst drew ROI over the right liver lobe on the wave image. The ROI is propagated to the elastogram for mean “shear stiffness” estimate (kPa). An automated 95% confidence grid (cross-hatched pattern within the ROI) indicates unreliable data that do not contribute to the mean stiffness estimate on the elastogram.

**Diagnostic performance of SWE and MRE at classifying dichotomized fibrosis stages for predefined sensitivity ≥ 90%, with intention-to-diagnose analysis including 3 failed MRE exams and 1 failed SWE exam in worst-case scenario (every failed exam counts as a false negative result when calculating sensitivity, and a false positive result when calculating specificity)**[8]**.**

| Fibrosis stage | Method | Cutoff | Sensitivity | Specificity |
| --- | --- | --- | --- | --- |
| Stage 1-4 vs 0 | MRE | 2.01 kPa | 0.867 | 0.457 |
|  | SWE | 1.27 m/s | 0.897 | 0.114 |
| Stage 2-4 vs 0-1 | MRE | 2.77 kPa | 0.792 | 0.817 |
|  | SWE | 1.49 m/s | 0.863 | 0.425 |
| Stage 3-4 vs 0-2 | MRE | 2.77 kPa | 0.789 | 0.782 |
|  | SWE | 1.46 m/s | 0.882 | 0.388 |
| Stage 4 vs 0-3 | MRE | 2.77 kPa | 0.667 | 0.711 |
|  | SWE | 1.59 m/s | 0.857 | 0.611 |

*MRE* magnetic resonance elastography; *SWE* shear wave elastography; *PPV* positive predictive value; *NPV* negative predictive value; *kPa* kilopascals, unit for shear stiffness as measured by MRE; *m/s* meters per second, unit for shear wave speed as measured by SWE

**Diagnostic performance of SWE and MRE at classifying dichotomized fibrosis stages for predefined specificity ≥ 90%, with intention-to-diagnose analysis including 3 failed MRE exams and 1 failed SWE exam in worst-case scenario (every failed exam counts as a false negative result when calculating sensitivity, and a false positive result when calculating specificity)**[8]**.**

| Fibrosis stage | Method | Cutoff | Sensitivity | Specificity |
| --- | --- | --- | --- | --- |
| Stage 1-4 vs 0 | MRE | 2.60 kPa | 0.550 | 0.848 |
|  | SWE | 1.75 m/s | 0.328 | 0.886 |
| Stage 2-4 vs 0-1 | MRE | 3.06 kPa | 0.708 | 0.878 |
|  | SWE | 1.79 m/s | 0.455 | 0.900 |
| Stage 3-4 vs 0-2 | MRE | 3.17 kPa | 0.684 | 0.874 |
|  | SWE | 1.78 m/s | 0.588 | 0.894 |
| Stage 4 vs 0-3 | MRE | 3.42 kPa | 0.444 | 0.876 |
|  | SWE | 1.81 m/s | 0.714 | 0.895 |

*MRE* magnetic resonance elastography; *SWE* shear wave elastography; *PPV* positive predictive value; *NPV* negative predictive value; *kPa* kilopascals, unit for shear stiffness as measured by MRE; *m/s* meters per second, unit for shear wave speed as measured by SWE

**References**

1. Vuppalanchi R, Ünalp A (2009) Increased Diagnostic Yield from Liver Biopsy in Suspected Nonalcoholic Fatty Liver Disease (NAFLD) Using Multiple Cores and Multiple Readings. Clin Gastroenterol Hepatol. https://doi.org/10.1016/j.cgh.2008.12.015.Increased

2. Dietrich C, Bamber J, Berzigotti A, et al (2017) EFSUMB Guidelines and Recommendations on the Clinical Use of Liver Ultrasound Elastography, Update 2017 (Long Version). Ultraschall der Medizin - Eur J Ultrasound 38:e16–e47. https://doi.org/10.1055/s-0043-103952

3. Jayakumar S, Middleton MS, Lawitz EJ, et al (2019) Longitudinal correlations between MRE, MRI-PDFF, and liver histology in patients with non-alcoholic steatohepatitis: Analysis of data from a phase II trial of selonsertib. J Hepatol 70:133–141. https://doi.org/10.1016/j.jhep.2018.09.024

4. Rockey DC, Caldwell SH, Goodman ZD, et al (2009) Liver biopsy. Hepatology 49:1017–1044. https://doi.org/10.1002/hep.22742

5. Kleiner DE, Brunt EM, Van Natta M, et al (2005) Design and validation of a histological scoring system for nonalcoholic fatty liver disease. Hepatology 41:1313–1321. https://doi.org/10.1002/hep.20701

6. Bydder M, Yokoo T, Hamilton G, et al (2008) Relaxation effects in the quantification of fat using gradient echo imaging. Magn Reson Imaging 26:347–359. https://doi.org/10.1016/J.MRI.2007.08.012

7. Hamilton G, Yokoo T, Bydder M, et al (2011) In vivo characterization of the liver fat 1H MR spectrum. NMR Biomed 24:784–790. https://doi.org/10.1002/nbm.1622

8. Schuetz GM, Schlattmann P, Dewey M (2012) Use of 3×2 tables with an intention to diagnose approach to assess clinical performance of diagnostic tests: meta-analytical evaluation of coronary CT angiography studies. BMJ 345:. https://doi.org/10.1136/BMJ.E6717
